# Fracture resistance of molars receiving nanoceramic-resin CAD/CAM onlays after cervical marginal elevation with different injectable restorative materials: effect of six-month water storage

**DOI:** 10.1007/s00784-025-06509-9

**Published:** 2025-08-18

**Authors:** Basema Nader Roshdy, Radwa Ibrahim Eltoukhy, Ashraf Ibrahim Ali, Salah Hasab Mahmoud

**Affiliations:** 1 Conservative Dentistry Department, Faculty of Dentistry, Horus University, New Damietta, Egypt; 2 Conservative Dentistry Department, Faculty of Dentistry, Mansoura University, 77 Al Gomhoria St, Mansoura, 35516 Egypt

**Keywords:** Onlay restorations, Cervical margin elevation, Injectable materials, Water storage, Bioactive materials, Fracture resistance

## Abstract

**Objective:**

To assess the influence of six-month water storage on the fracture resistance of molars receiving mesio-occluso-distal (MOD) nanoceramic CAD/CAM onlay restorations after cervical marginal elevation (CME) with different injectable restorative materials.

**Materials and methods:**

Two hundred ten sound mandibular molars received standardized MOD onlay preparations with cervical margins extending 2 mm apical to the cementoenamel junction (CEJ). Molars were randomly assigned into five groups (*n* = 42) according to the restorative materials used for CME: No-CME Group, control; CME-HVGI Group, highly viscous glass ionomer; CME-ICR Group, injectable composite resin; CME-RMGI Group, resin-modified glass ionomer; CME-BAIR Group, bioactive ionic resin. Immediate dentin sealing was performed on each molar before receiving nanoceramic-resin CAD/CAM onlay restoration. Each group was subdivided into two subgroups (*n* = 21) based on whether they underwent six months of water storage. All specimens were subjected to clinical simulation via thermo-mechanical loading, fracture resistance testing, and failure mode analysis.

**Results:**

No statistically significant difference in fracture resistance was observed when comparing the tested CME groups to the control group after six months of water storage. Regarding failure mode, irreparable failure was significantly dominant, with no significant difference among all groups.

**Conclusions:**

Six-month water storage had no adverse effect on the fracture resistance of teeth receiving nanoceramic onlay restorations, regardless of the type of cervical margin elevating material used.

**Clinical relevance:**

The smoothly handled injectable restorative materials used for CME of molars with nanoceramic-resin CAD/CAM onlay restorations could endure humid conditions, exhibiting acceptable performance under compressive loading.

## Introduction

Restoring extensive proximal carious lesions, particularly those extending subgingivally, poses a significant clinical challenge. Advances in adhesive dentistry and CAD/CAM technologies have led to various restorative approaches, with an increasing preference for indirect nanoceramic onlay restorations. These restorations provide superior esthetics and mechanical strength, along with considerable resiliency and biomimetic wear characteristics, reinforcing weakened tooth cusps. Nevertheless, achieving durable restoration for deep cervical cavity preparations remains a critical concern [1–6].

Cervical margin elevation has been introduced as a minimally invasive technique to overcome the challenges of managing subgingival margins. This procedure involves repositioning the cervical margin to a manageable supragingival position using direct restorative materials, thereby facilitating improved isolation, impression accuracy, and cementation procedures. Furthermore, by reducing the occluso-cervical height of the proximal boxes, CME may decrease the risk of fracture-induced failures [7–10].

While offering clinical advantages, the restorative materials used in this technique have raised concerns. Injectable restorative materials provide improved adaptation to irregular surfaces, simplifying restorative procedures [11–13]. Recent generations of injectable resin composites are praised for their favorable aesthetic and mechanical properties [14, 15]. Conversely, glass ionomer cements exhibit excellent biocompatibility and fluoride-releasing capabilities, which may reduce the risk of secondary caries. Resin-modified glass ionomers and recently developed bioactive ionic resins have evolved to enhance the mechanical properties of glass ionomer cement [16–18].

Given the clinical prevalence of fracture as a cause of indirect restoration failure [19, 20], a related study [21] examined the effect of CME with different injectable restorative systems on the fracture resistance of molars restored with CAD/CAM nanoceramic onlay restorations. It revealed a negligible impact of CME on the fracture resistance of the studied indirect restorative systems. However, the hydrolytic degradation potential of the oral environment underscores the importance of evaluating the long-term stability of these restorations. Water storage can significantly affect the mechanical properties of several restorative materials, potentially influencing the overall fracture resistance of the tooth-restoration system [17, 22]. Therefore, this study evaluated the fracture resistance of molars receiving CAD/CAM onlays with CME using various injectable restorative materials after six months of water storage, utilizing the related study groups [21] as parallel no-storage controls. The null hypothesis assumed that six-month water storage would not affect the fracture resistance of nanoceramic onlay restorations preceded by CME.

## Materials and methods

### Materials

Specifications, compositions, and lot numbers of the restorative materials used in this study are detailed in Table 1.

**Table 1:** Restorative materials used in this study

Material	Specification	Composition	Manufacturer	Lot number
Equia Fil	Highly-viscous glass ionomer	Powder: Fluoro_alumino_silicate glass Liquid: Polyacrylic acid, polybasic-carboxylic acid, distilled water	GC, Tokyo, Japan	2,204,061
G-aenial Universal Injectable	Highly-filled injectable composite resin	Resin matrix: UDMA, TEGDMA, Bis-MEPP Filler: 69 wt %; Silica-barium glass	GC, Tokyo, Japan	2,108,191
GC Fuji II LC	Resin-modified glass ionomer	Powder: Alumino-fluoro- silicate glass Liquid: Polyacrylic acid, HEMA, Trimethyl hexamethylene dicarbonate, UDMA, TEGDMA	GC, Tokyo, Japan	2,306,201
Activa Bioactive Restorative	Bioactive ionic-resin	Blend of diurethane and other methacrylates with modified polyacrylic acid, patented rubberized-resin (Embrace), reactive glass filler, inorganic filler, sodium fluoride, water	Pulpdent, Watertown, MA, USA	210,402
G-Premio BOND	Universal adhesive	10-MDP, 4-MET, MEPS, Acetone, Water, Silica	GC, Tokyo, Japan	2,205,301
Grandio Blocs	Nanoceramic-resin CAD/CAM blocks	86 wt % nano-hybrid fillers Barium-alumino-borosilicate glass, SiO_2_, UDMA, DMA	VOCO, Cuxhaven, Germany	2,050,571
Bifix SE	Dual- cured, self- adhesive resin-cement	Catalyst: phosphoric acid, dimethacrylates, dimethacrylate ester, methacrylates, BPO, SiO2, BAS glass ceramic, BHT Base: dimethacrylates, methacrylates, CQ, amine, SiO2, BAS-glass ceramic, BHT; 66.3% filler content	VOCO, Cuxhaven, Germany	2,131,229

UDMA, urethane dimethacrylate; TEGDMA, triethylene glycol dimethacrylate; Bis-MEPP, 2,2-bis-(4-methacryloxy polyethoxy) phenyl propane; HEMA, hydroxy ethyl methacrylate; 10-MDP, 10-methacryloyloxydecyl dihydrogen phosphate; 4-MET, 4-methacryloxyethyl trimellitic acid; MEPS, methacryloyloxyalkyl thiophosphate methylmethacrylate; DMA, dimethacrylate; BPO, benzoyl peroxide; BHT, butylated hydroxytoluene; CQ, camphorquinone

### Ethical approval and specimen collection

This study received approval from the Dental Research Ethics Committee, Mansoura University (ethical approval number: M10060721). Sound, freshly extracted mandibular molars were obtained from medically healthy individuals aged 45–55 undergoing extractions due to periodontal disease. All participants provided informed consent. Following extraction, molars were cleaned and disinfected in a 0.5% chloramine-T solution for 72 h. Crack-free molars were selected and examined under a stereomicroscope (30×, Olympus SZ TP) and exhibited comparable dimensions, with intercuspal distances ranging from 5.2 to 6.1 mm. The teeth were stored in distilled water at 4 °C for a maximum of three months until use.

### Sample siza calculation

The sample size was calculated using G*Power software, version 3.1.9.7, developed by Universität Kiel, Germany. ANOVA: fixed effects, special, main effects, and interactions were selected, with 0.80 power, medium effect size (f = 0.25), ten groups, and numerator degrees of freedom as (5 − 1) × (2 − 1) = 4. This calculation yielded a sample size of 200 teeth. Two hundred ten specimens were included, providing one extra molar specimen per group.

### Study design and randomization

**Fig. 1:**
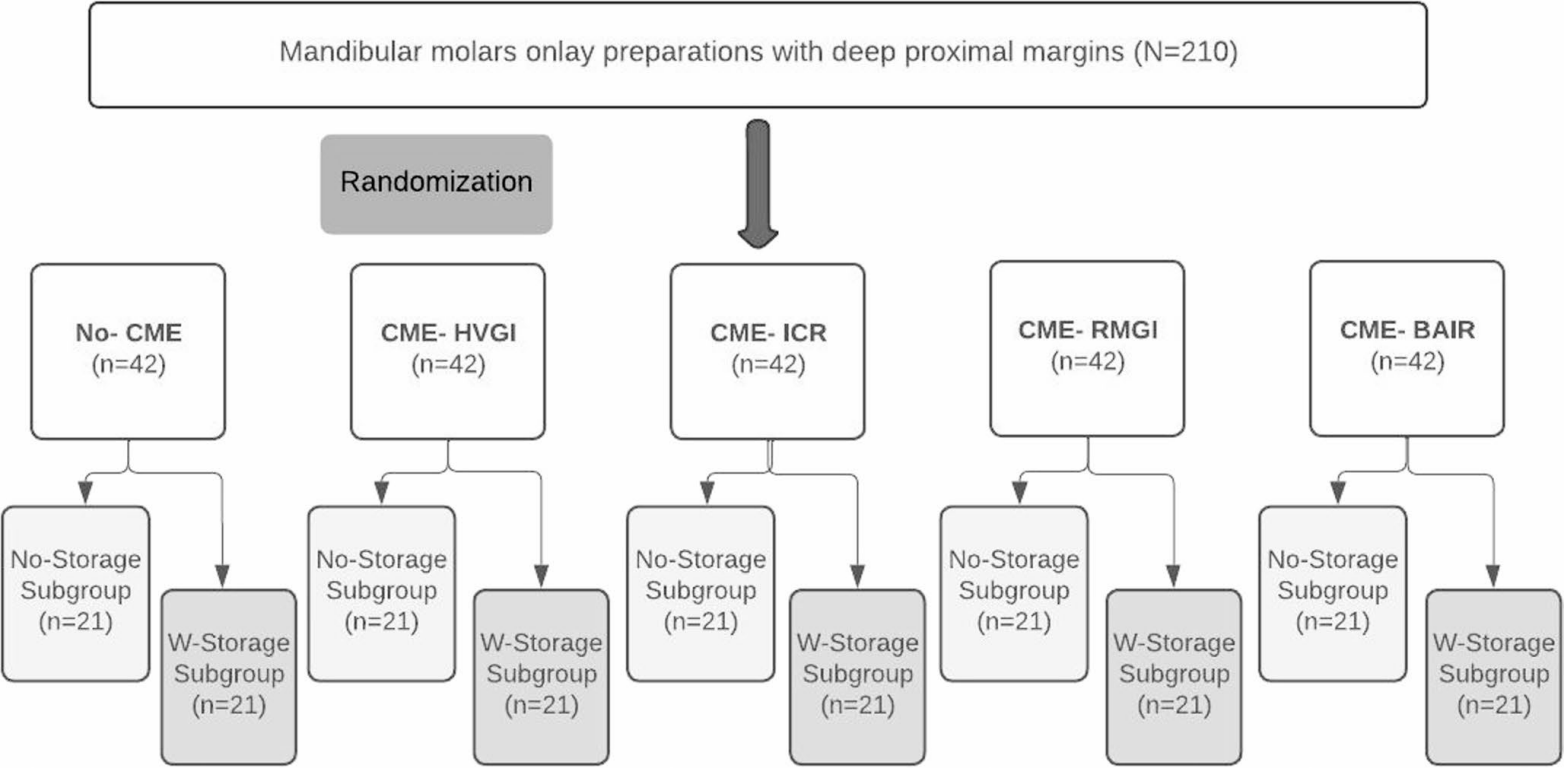
Study design and grouping

The study was designed with five groups (*n* = 42) based on the CME material used. Each group was further subdivided into two subgroups (*n* = 21): no-storage subgroup and water-storage (W-storage) subgroup, as illustrated in Fig. 1. Randomization of teeth was performed using IBM SPSS (version 26, Armonk, NY, USA).

### Onlay preparation

Polyvinyl chloride rings (1.4 cm in diameter and 2 cm in length) were used to embed teeth in self-curing acrylic resin up to 3 mm apical to CEJ. A 0.3 mm layer of polyvinyl siloxane impression material (V-Posil Light Fast; VOCO, Cuxhaven, Germany) was applied using the lost wax technique [23]. Throughout the preparation, teeth were maintained in distilled water to prevent dehydration.

**Fig. 2:**
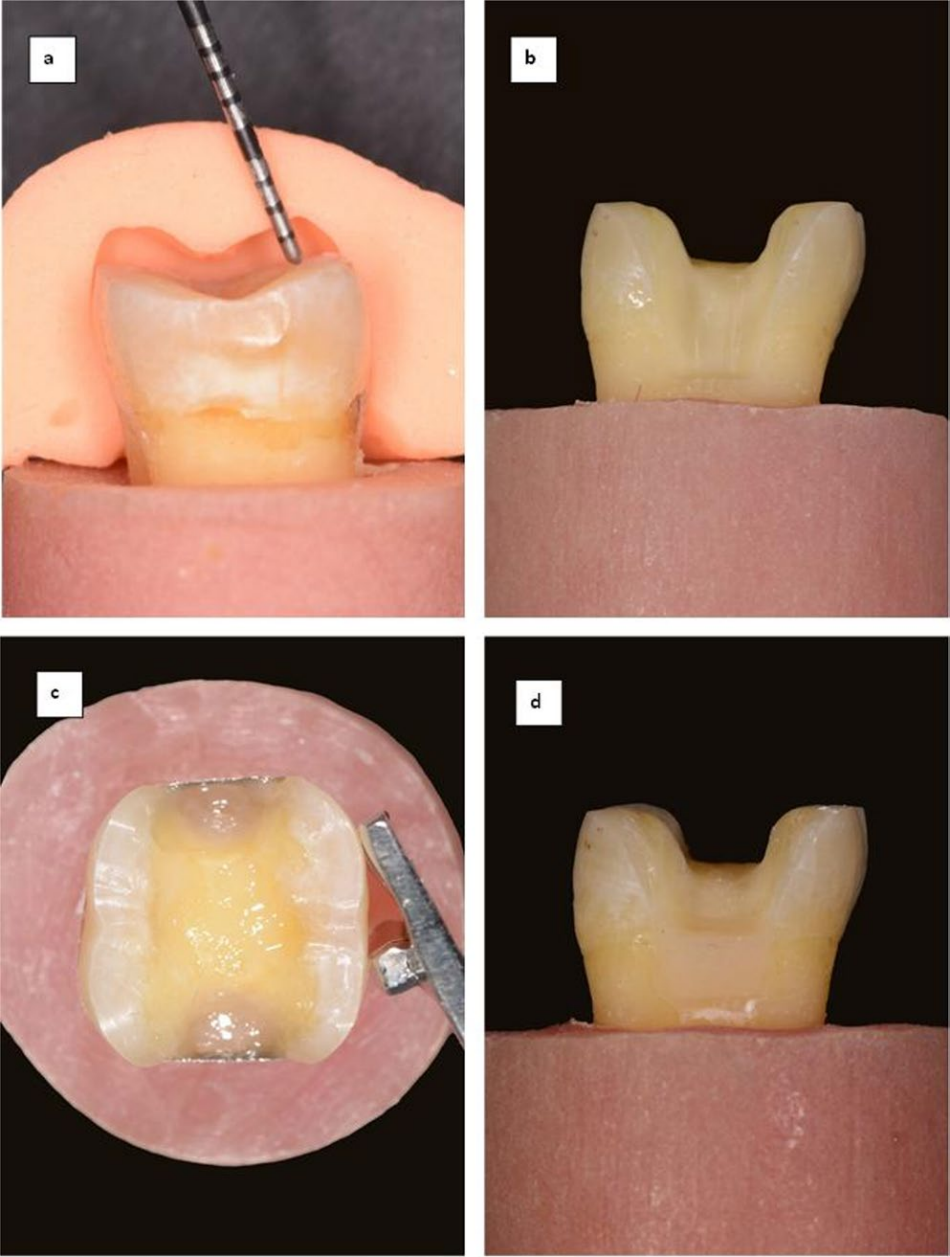
**(a)** Checking occlusal reduction using rubber base index **(b)** Tooth preparation for onlay restoration. **(c)** cervical margin elevation with adapted modified matrix band **(d)** Tooth preparation after cervical margin elevation

Each tooth underwent preoperative formation of a putty rubber base index to ensure standardized preparation, followed by digital scanning to guide the design of the onlay restoration. One experienced operator prepared standardized MOD cavities using tapered diamond points #8117 and #8113NR (Inlay Set, Intensiv, Viganello-Lugano, Switzerland) and a high-speed handpiece under water cooling. Occlusal part dimensions were 3 mm in depth and buccolingual width. Proximal boxes extended 2 mm apical to the CEJ, with a buccolingual width of 3 mm apically and 5 mm occlusally, and an axial depth of 1.5 mm [24]. All cusps were anatomically reduced to a depth of 1.5 mm, with a butt joint cavosurface angle. Depth cuts and the rubber index ensured consistent cusp reduction (Fig. 2a). Cavity dimensions were marked on the tooth surfaces using a digital caliper and marker. Depth was verified with a periodontal probe under 3.5× magnification loupes (Rose Micro Solutions, West Seneca, New York). Preparation walls were finished to establish smooth surfaces and rounded line angles (Fig. 2b) [21].

### Cervical margin elevation

The modified matrix band technique was used to apply a 2 mm thickness of CME restorative materials up to the CEJ. The middle part of the Tofflemire matrix bands was trimmed to 3 mm in height to facilitate adaptation to the cervical margins (Fig. 2c). CME steps were performed according to the assigned group, following the manufacturer’s recommendations [21]:

- **No-CME Group**: Freshly prepared dentin only was sealed with a universal adhesive, followed by a thin layer of flowable composite.
- **CME-HVGI Group**: Cervical margins were conditioned with dentin conditioner (GC, Tokyo, Japan) before applying the highly viscous glass ionomer up to the CEJ. Subsequently, exposed dentin was sealed with the universal adhesive. Then, the external surface of HVGI was resin-coated with EQUIA Forte^®^ Coat (GC Co, Tokyo, Japan).
- **CME-ICR Group**: A universal adhesive was applied to the cervical seats and exposed dentin. An injectable composite was applied cervically and light-cured, followed by a thin layer that supported sealed dentin surfaces.
- **CME-RMGI Group**: CME and dentin sealing were performed as in CME-HVGI Group, differing only by the application and light-curing of RMGI.
- **CME-BAIR Group**: A universal adhesive was applied to the cervical seats and exposed dentin, followed by BAIR deposition at the proximal cervical seats and light curing.

Great care was taken to avoid air bubbles. Specimens from all groups underwent additional light-curing with a glycerine gel layer to prevent a polymerization-inhibition layer. Enamel margins were refined using white color-coded diamond abrasives, No. 3113R (Inlay Set, Intensiv, Viganello-Lugano, Switzerland). A representative photograph for the tooth preparation after CME is shown in Fig. 2d.

### Restorations' fabrication and cementation

Provisional restorations were fabricated and cemented using eugenol-free temporary cement (Provicol; VOCO, Cuxhaven, Germany) until the final restoration was completed. Final indirect restorations were fabricated according to the manufacturer’s instructions. Restoration fitting surfaces were sandblasted with 50-µm aluminum oxide particles and cleaned in an ultrasonic bath.

Temporary cement residues were carefully removed using a scaler. Airborne-particle abrasion with silicated aluminum oxide (CoJet Sand; 3 M ESPE, Deutschland, Germany) was performed for 4 s at a pressure of 2 bar, using a 45° nozzle angle and a 10 mm distance. Silanization was performed with a coupling agent (Ceramic Bond, VOCO, Cuxhaven, Germany) for 60 s, followed by air drying. Enamel margins were selectively etched with 37% phosphoric acid (Ivoclar Vivadent, NY, USA) for 30 s and rinsed [24, 25]. Onlay restorations were cemented using self-adhesive resin cement under a constant seating load of 3 kg [26].

### Specimens' treatment

Specimens in the W-storage subgroups were stored in distilled water in an incubator (Model 20GC, Quincy Lab, Chicago, IL, USA) at 37 °C for 6 months. Subsequently, all specimens were subjected to thermo-mechanical loading to simulate clinical conditions. Mechanical loading was performed using a ROBOTA chewing simulator (Model ACH-09075DC-T, AD-Tech Technology Co., Ltd., Germany). A chewing force of 49 N was applied at a frequency of 1.6 Hz for 150,000 cycles, with a 40 mm/s descending and backward speed and a torque of 2.4 N·m [27]. Specimens underwent 10,000 thermal cycles between 55 °C and 5 °C, with a 25-second dwell time and a 10-second lag time using Robota automated thermal cycle (BILGE, Turkey) [28].

### Fracture resistance test

Specimens were mounted on a materials testing machine (Model 3345; Instron Industrial Products, Norwood, MA, USA) with a 5 kN load cell, and data were recorded using computer software (Bluehill Lite Software, Instron^®^). The long axis of the tooth was lingually oriented with a 15-degree inclination. A compressive load was applied at the central fossa at a speed of 1 mm/min, using a 4.8 mm diameter spherical-tipped metallic rod and a separating tin foil sheet. The load at failure manifested by an audible crack and confirmed by a sharp drop in the load-deflection curve recorded by the software. Failure load was recorded, and failure modes were determined using 25 X stereomicroscope (SZ TP, Olympus, Tokyo, Japan). Failure modes were categorized as reparable (restoration/coronal fracture) or irreparable (fracture below CEJ).

### Statistical analysis

The assembled data were analyzed using the Statistical Package for the Social Sciences (IBM SPSS, version 26, Armonk, NY, USA). Fracture resistance data were assessed for normality using the Kolmogorov-Smirnov test. Two-way analysis of variance (ANOVA) and Tukey’s tests were used for statistical analysis. Failure modes were evaluated using the Pearson chi-square (X^2^) test. The level of significance was set at *p* ≤ 0.05.

## Results

Table 2 displays mean values and standard deviations of fracture resistance. Analysis of the interaction between the two study variables (CME material and water-storage factors) using two-way ANOVA showed a non-significant interaction effect on fracture resistance (F = 0.163, *P* = 0.96). Individual factor analysis revealed a negligible effect of the water-storage factor (*P* = 0.28) and a significant influence of CME material (*P* < 0.001).

**Table 2:** Results of fracture resistance

Fracture resistance						
Group	Subgroup					
	No-water storage			Water storage		
**Group**	Mean	St. Dev.	Sig.**	Mean	St. Dev.	Sig.**
**NO-CME**	1853.7	347	A, B	1813.7	307	A, B
**CME-HVGI**	1778.4	280	A, B	1762.9	230	A, B
**CME-ICR**	2054.2	386	A	2036.6	330	A
**CME-RMGI**	1803.5	343	A, B	1689.5	276	B
**CME-BAIR**	1832.0	263	A, B	1783.2	348	A, B
P*	*P* = 0.96					

* *P* values resulted from two-way ANOVA test

** Subgroups having the same letter are not significantly different

*P* < 0.05 denotes significant difference

Post hoc Tukey’s tests showed no significant differences in the mean values of fracture resistance between the W-storage CME subgroups individually and the control No-CME Group. Similarly, comparisons between No-storage and W-storage subgroups within each group revealed no significant differences. Multiple comparisons among fracture resistance mean values of different CME W-storage subgroups revealed significantly higher fracture resistance in the W-storage subgroup of CME-ICR Group compared to CME-RMGI Group. Failure mode results showed no statistically significant differences (X^2^ = 3.13, *P* = 0.96). Irreparable fracture modes were significantly more prevalent than reparable failure modes in all test groups (Table 3).

**Table 3:** Analysis of failure mode of the studied groups

Group		NO-CME	CME-HVGI	CME-ICR	CME-RMGI	CME-BAIR	Test of significance
Failure Mode							
No-storage Subgroup	Repairable	2	4	2	2	3	X^2^ = 1.4 *P* = 0.84
	Irreparable	19	17	19	19	18	
W-storage Subgroup	Repairable	4	3	2	4	2	X^2^ = 1.6 *P* = 0.82
	Irreparable	17	18	19	17	19	
Test of significance		X^2^ = 0.78 *P* = 0.38	X^2^ = 0.17 *P* = 0.68	X^2^ = 0.00 *P* = 1	X^2^ = 0.78 *P* = 0.38	X^2^ = 0.23 *P* = 0.63	X^2^ = 3.13 *P* = 0.96

X^2^ = Chi-Square test

*P* < 0.05 denotes significant difference

## Discussion

The optimal restorative approach for managing extensive subgingival proximal caries in posterior teeth remains a topic of debate. Adhering to minimally invasive principles, the application of CME material beneath partial indirect esthetic restorations has been recommended [29]. Considering the hydrolytic nature of the oral cavity, which can adversely affect the characteristics and longevity of restorative materials, this study assessed the influence of six months of water immersion on the fracture resistance of various injectable CME materials applied to molars featuring CAD/CAM nanoceramic onlay restorations. The previously designed study [21], which investigated the effect of CME with different injectable restorative materials, was employed as a control, using a similar methodology in parallel groups with no storage.

The control no-water storage grouping enabled the investigation of the effect of CME using various injectable materials, as previously explained [21]. Comparing the no-water storage groups to parallel water-storage groups facilitates the determination of the impact of water storage on different restorative systems. The methodology of this study paid close attention to clinical simulation. Periodontal ligament and bone support are vital for stress distribution mechanisms in teeth. For fracture resistance testing, the root embedment material was used to simulate bone and support the tooth during compressive loading, while an elastomeric material was employed to replicate the clinical force distribution more accurately [30, 31]. Specimen teeth received provisional restorations until the fabrication of final restorations, thereby mimicking clinical scenarios. This protected the dentin-sealed surfaces and reflected the steps for surface preparation and cementation. Moreover, all specimens underwent mechanical loading and thermal cycling to predict probable clinical failure [27]. Finally, during the fracture resistance test, a compressive load was applied at 15 ° to the tooth’s long axis to simulate the direction of occlusal load intraorally.

The study’s findings support accepting the null hypothesis. Six months of water storage did not affect the fracture resistance of the tested restorative systems with elevated cervical margins. None of the evaluated CME material groups showed a significant impact of six-month water storage on their restorative system’s resistance to compressive stresses. Meanwhile, CME-ICR exhibited better fracture resistance than CME-RMGI after water storage.

Storage has been noted to have varying effects on restorative materials [17, 22, 32]. Since water sorption of a given polymer is influenced by its hydrophilicity [33], the increased hydrophilicity and water sorption of glass ionomer-based materials could explain decreased strength compared to resin composite after aging [22]. However, HVGI and RMGI materials have been reported to show lower water sorption and improved mechanical strength with resin coating [34–36]. This likely accounts for the negligible effect of water storage on their performance under compressive stresses.

The superior performance of the highly filled injectable composite can be attributed to its favorable solubility and water sorption properties [37], which enable it to retain its strength after water aging [38]. Moreover, highly filled injectable resin has been reported to reduce stresses on the cervical margin area [14, 15]. Flowable lining dental materials with low elastic modulus may provide a stress-relieving effect by absorbing stresses to withstand forces. However, the lower mechanical strength of modified glass ionomers relative to resin composites may inadequately compensate for compressive stresses, explaining their lower resistance under such stresses [12, 39].

Failure modes were predominantly irreparable across all tested groups, consistent with other studies [19, 21, 24, 40] that report a high incidence of catastrophic failures associated with onlay systems. Onlay systems after CME, which restore subgingival cavities, have been reported to exhibit more fractures below the CEJ, alongside increased fracture resistance values [24]. All restorations demonstrated greater fracture resistance than the average intraoral force exerted during mastication. Overall, the results of this study support elevating subgingival cervical margins with injectable restorative materials as a CME preceding CAD/CAM onlay restorations. Despite efforts to simulate clinical conditions, precise clinical replication in vitro is limited due to various oral cavity parameters that challenge restorative systems.

## Conclusion

Considering the limitations of this in vitro study, it can be implied that six months of water storage exhibited no adverse effect on the fracture resistance of molars restored with nanoceramic CAD/CAM onlay restorations. Cervical margin elevation using injectable resin composite demonstrated better performance under compressive loading compared to resin-modified glass ionomer after six months of water storage.

## Data Availability

The datasets used and/or analyzed during the current study are available from the corresponding author on reasonable request.
